# Elucidating the Implications of Norovirus *N*- and *O*-Glycosylation, *O*-GlcNAcylation, and Phosphorylation

**DOI:** 10.3390/v15030798

**Published:** 2023-03-21

**Authors:** Chia-Chi Cheng, Guan-Ming Ke, Pei-Yu Chu, Liang-Yin Ke

**Affiliations:** 1 Department of Medical Laboratory Science and Biotechnology, College of Health Sciences, Kaohsiung Medical University, Kaohsiung 807378, Taiwan; 2 Graduate Institute of Animal Vaccine Technology, College of Veterinary Medicine, National Pingtung University of Science and Technology, Pingtung 912301, Taiwan; kegm@mail.npust.edu.tw; 3 Center for Lipid Biosciences, Department of Medical Research, Kaohsiung Medical University Hospital, Kaohsiung 807378, Taiwan; 4 Graduate Institute of Medicine, College of Medicine, Kaohsiung Medical University, Kaohsiung 807378, Taiwan

**Keywords:** norovirus, *N*- and *O*-glycosylation, *O*-GlcNAcylation, phosphorylation

## Abstract

Norovirus is the most common cause of foodborne gastroenteritis, affecting millions of people worldwide annually. Among the ten genotypes (GI–GX) of norovirus, only GI, GII, GIV, GVIII, and GIX infect humans. Some genotypes reportedly exhibit post-translational modifications (PTMs), including *N*- and *O*-glycosylation, *O*-GlcNAcylation, and phosphorylation, in their viral antigens. PTMs have been linked to increased viral genome replication, viral particle release, and virulence. Owing to breakthroughs in mass spectrometry (MS) technologies, more PTMs have been discovered in recent years and have contributed significantly to preventing and treating infectious diseases. However, the mechanisms by which PTMs act on noroviruses remain poorly understood. In this section, we outline the current knowledge of the three common types of PTM and investigate their impact on norovirus pathogenesis. Moreover, we summarize the strategies and techniques for the identification of PTMs.

## 1. Introduction

The most prevalent causes of foodborne outbreaks are noroviruses, which account for approximately 50% of all occurrences worldwide [1]. They cause 20% of all gastrointestinal illnesses worldwide, resulting in 200,000 deaths and 700 million infections annually [2,3]. Norovirus infections are characterized by emesis, acute watery diarrhea, nausea, low-grade fever, and abdominal cramps. Norovirus infections are usually transient, with symptoms vanishing within 12–72 h [4]. Although norovirus-associated disease is usually self-limiting, exposure to norovirus leaves neonates, the elderly, and immunocompromised patients vulnerable to chronic severe or life-threatening symptoms [5,6]. Norovirus infections are common among people of all ages and cause substantial health and economic burdens in developed and developing countries [6].

Noroviruses are single-stranded positive-sense ribonucleic acid (RNA) viruses that belong to the *Caliciviridae* family. Genomic RNA is covalently coupled to a viral protein (VPg) at the 5′ end and polyadenylated at the 3′ end [7]. Most norovirus genomes are structured into three open reading frames (ORFs), whereas murine noroviruses have four ORFs [8]. ORF1 encodes nonstructural proteins NS1/2 to NS7. Among these, NS7, an RNA-dependent RNA polymerase (RdRp), plays a crucial role in genome replication. ORF2 encodes the major capsid (VP1), which has a shell (S) and protruding (P) domain. The P domain can be divided into P1 and P2 subdomains. P2 can recognize human histo-blood group antigens (HBGAs), which are essential for receptor binding [9,10,11]. ORF3 encodes minor capsid proteins (VP2), which are involved in capsid assembly and genome encapsidation [7,8,12]. The genetic classification of noroviruses is based on VP1 and RdRp [7,8]. There are 10 genogroups (GI–GX) and 49 norovirus genotypes, among which GII genotype 4 (GII.4) is responsible for most norovirus outbreaks worldwide [8].

Previously, research into human norovirus (HuNoV) pathogenesis has been hampered because it could not be propagated successfully in cell culture [5]. Murine norovirus (MNV) and feline calicivirus (FCV) have been utilized as surrogates to circumvent this barrier [6,13] owing to their shared genetic and biochemical features with HuNoV [6,14]. According to recent research, HuNoV and FCV have post-translational modifications (PTMs) in their RdRp, VPg, and P domains [11,15,16]. Moreover, human intestinal enteroids (HIEs) generated from stem cells and zebrafish larvae are effective HuNoV replication models [17,18,19,20]. These systems have the potential to give a better understanding of HuNoV biology, pathophysiology, and underlying mechanisms of infection and PTMs. Infections in HIE cultures indicate the link between HBGA glycosylation and norovirus infection [21]. On positive-sense RNA viruses, modification of the cellular translational apparatus occurs, which is thought to be favorable for viral infection [16,22]. However, knowledge of norovirus modifications has not yet been systematically organized.

Thus, we outline the current understanding of PTMs and explore their influence on norovirus pathogenesis. We comprehensively discuss in particular the mechanisms by which phosphorylation, *N*- and *O*-glycosylation, and *O*-GlcNAcylation affect norovirus pathogenesis and raise the issue of a lack of information on modifications of noroviruses.

## 2. Post-Translational Modifications

### 2.1. Effects of Post-Translational Modifications on Protein Function

Protein PTMs provide crucial insights into various cellular functions [23]. PTMs are typically formed by enzymatic processes that add functional groups to the side chains of amino acids [24]. These modifications are reversible and essential for biological functions. Over 620 modifications have been discovered that occur after protein synthesis [25]. *N*- and *O*-glycosylation, *O*-GlcNAcylation, and phosphorylation are the most common modifications that increase protein solubility, conformation, interactions, signaling, and degradation, which are all critical for cell growth [26]. *N*- and *O*-glycosylation facilitates receptor binding and alters the structure of the secreted proteins [27,28]. In contrast, *O*-GlcNAcylation and phosphorylation are competitive processes implicated in many signal transduction pathways [29,30].

### 2.2. Effects of Post-Translational Modifications on Viruses

Many pathogens, including viruses and bacteria, can utilize post-translational modifications to enhance interactions with host proteins crucial to infection (Table 1). As viruses rely on the protein synthesis machinery of host cells to support their replication, most viral proteins are subjected to PTMs [31]. This improves viral replication, assembly, release, and immune escape during infection, thereby promoting virus propagation. Furthermore, PTMs improve solubility and antigenicity, which enhances virulence [29]. For example, phosphorylation of the dengue virus (DENV) type 2 regulates interactions between viral replication proteins [22]. In this review, we elucidate the effects of PTMs on noroviruses. Here, we focus on the mechanism of *N*- and *O*-glycosylation, *O*-GlcNAcylation, and phosphorylation and discuss the mechanisms by which these modifications affect norovirus pathogenesis.

### 2.3. Identification of Post-Translational Modifications

Previously, studies on PTMs were limited owing to the requirement for laborious biochemical approaches, including radioactive-isotope-labeled substrates, antibody-based Western blot analysis, and peptide and protein arrays [57,58,59]. However, these procedures are inefficient owing to the difficulty in identifying modified proteins using their corresponding weakly radioactive-isotope-labeled substrates. Furthermore, creating antibodies that recognize the minor structural motifs of particular PTMs using antibody-based Western blot analysis is also challenging [59]. Over the last decade, mass spectrometry (MS) has been demonstrated to be a powerful technique for identifying modified proteins and mapping PTM locations. Sites containing *N*- and *O*-glycosylation, *O*-GlcNAcylation, and phosphorylation modifications were enriched and successfully identified using liquid chromatography-tandem mass spectrometry (LC-MS/MS) [59,60,61]. In addition, enzymes or inhibitors may be used to investigate PTMs (Table 1). Section 3.1, Section 4.1 and Section 5.1 in this article comprehensively describe these mechanisms.

## 3. *N*- and *O*-Glycosylation

### 3.1. N- and O-Glycosylation on Proteins

**Figure 1:**
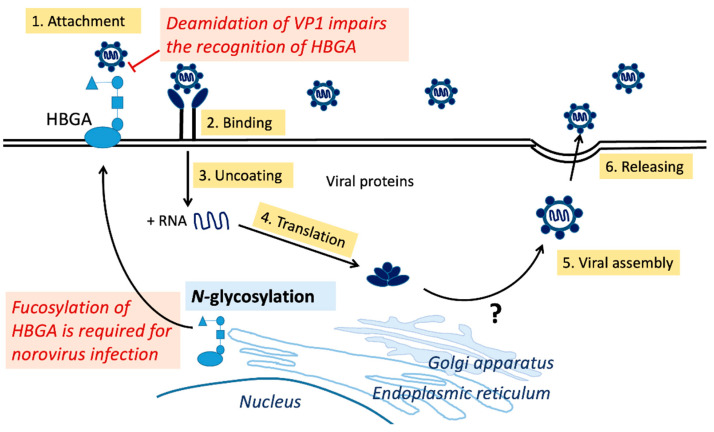
Replication cycle of noroviruses and protein post-translational modifications. (1) Attachment: Human norovirus (HuNoV) attaches to the HBGAs on the host cell surface, allowing viral entrance. Note that fucosylation of HBGA by fucosyltransferase 2 (FUT2) is required for certain genotypes of norovirus infection. In contrast, deamination on Asn373 of norovirus capsid protein VP1 impairs the recognition of HBGAs. (2) Binding. (3) Uncoating through undefined pathways [80]. (4) Translation: The positive-sense RNA genome may serve as a template for viral protein translation. After translation, viral proteins could undergo *N*- or *O*-glycosylation at the endoplasmic reticulum or Golgi apparatus. Mechanisms of noroviral protein glycosylation remain unclear. (5) Assembly: viral proteins assemble to form new viral particles. (6) Release: viral particle release from host cell [81]. Abbreviations: HBGA, histo-blood group antigen.

Glycosylation is a multienzymatic process that produces various glycoconjugates covalently bound to lipids or proteins [62]. Glycosylation is classified as *N*-linked or *O*-linked [30] (Figure 1). The first step in *N*-linked glycosylation is the attachment of an *N*-linked glycan to the asparagine residue of a nascent polypeptide chain by oligosaccharyltransferase (OST) in the endoplasmic reticulum (ER). Other enzymes subsequently construct glycans, resulting in a diverse spectrum of glycan structures such as oligomannose, hybrid, and complex-type *N*-glycan structures [30,45,62]. In *O*-linked glycosylation, *N*-acetylgalactosamine (GalNAc) is covalently linked to the hydroxyl group of serine or threonine residues in the Golgi apparatus [45,63]. This process is mediated by 20 different GalNAc transferases, each of which may produce unique mucin-type *O*-glycan core structures [45]. *N*- and *O*-glycosylation can alter protein characteristics, such as stability, solubility, protease resistance, and biological activity [64]. For example, glycans can be structurally integrated into the protein fold and exhibit significant glycan–protein interactions to stabilize the protein [30].

Traditional *N*-glycosylation detection methods include mutagenesis of anticipated glycosylation sites and enzymes to cleave glycans from protein substrates, which aids in distinguishing between terminally and core-glycosylated *N*-glycans. The peptide-*N*-glycosidase F (PNGase F) specifically cleaves the linkage between the innermost GlcNAc and asparagine. In addition, endoglycosidase H cleaves within the chitobiose core of high mannose and some hybrid oligosaccharides from *N*-linked glycoproteins. The molecular weight and functionalities of an *N*-glyco protein can be altered after incubation with these *N*-glycan removal enzymes (Table 1). Tunicamycin, for example, acts as an analog of uridine diphosphate *N*-acetylglucosamine (UDP-GlcNAc) to inhibit dolichol phosphate-dependent *N*-acetylglucosamine 1-phospho-transferase (DPAGT1), thus preventing the first step in *N*-glycoprotein biosynthesis [65]. In addition to the approaches stated above, gel electrophoresis and immunoblotting with antibodies are practical since PTM with glycan moieties changes the electrophoretic mobility of the protein [63].

Enrichment and MS technologies are used in modern strategies for detecting glycoproteins. Most *N*-glycoprotein enrichment procedures are based on hydrazide chemistry, which involves oxidation of the carbohydrate side chain and conjugating glycopeptides to hydrazide resin [66]. The isolated glycopeptides are then released using a glycan-specific enzyme, such as peptide-*N*-glycosidase F (PNGase F), followed by MS identification, thereby facilitating a comprehensive analysis of the *N*-glycosylated proteome [59,63,67]. Using the GlycoStore database, approximately 850 unique glycan structures of glycoproteins and glycolipids can be determined [68]. Alternatively, the use of data-independent collection mode mass spectrometry (MS^E^) and ProteinLynx Global Server (PLGS) software (Waters Corporation, Milford, MA, USA) may help identify short glycopeptides [28,69]. As for the identification of complicated *N*-glycans, PNGase F can liberate glycans and subsequently label them with the fluorophore 2-aminobenzoic acid (2-AA). The 2-AA tagged glycans can be purified by solid-phase extraction and detected using a fluorescence detector or mass spectrometer [70].

### 3.2. N- and O-Glycosylation on Viruses

Flaviviruses, severe acute respiratory syndrome-associated coronavirus (SARS-CoV2), influenza viruses, and rotaviruses exhibit viral protein *N*- and *O*-glycosylation (Table 1), which aids in viral entry, assembly, transmission potential, virulence, and pathogenicity [30]. RNA viruses manufacture their envelopes and surface glycoproteins using the host ER/Golgi system. Additionally, *N*-linked glycans can facilitate the folding and trafficking of viral glycoproteins via host ER quality control [71]. Viruses are often highly glycosylated on their surfaces, which increases the attachment of viral proteins to cells and facilitates infection [64]. Furthermore, they can mask or modify antibody-mediated recognition of antigenic epitopes, helping them evade the immune system of the host [64,71].

For example, glycosylation of the DENV NS1 aids in protein secretion by forming hexamers that bind to lectin pathway proteins such as C1s, C4, C4b-binding protein (C4BP), and mannose-binding lectin (MBL). This modification assists immune evasion by limiting lectin complement activation and DENV neutralization, controlling pathogenesis, and contributing to virulence [30,36,72]. In SARS-CoV-2, the spike protein, as well as M and E proteins, are glycosylated and responsible for membrane fusion, invasion, and immune escape [35,43]. The spike protein is attached by *N*-glycan, which facilitates its entry into the host cells and protects the epitopes to evade the immune response [43,44]. *N*-linked and *O*-linked glycosylation of the M protein facilitates viral particle assembly and budding. The E protein is involved in many viral processes, including membrane construction and interactions with other membrane proteins, and has two glycosylation sites, N48 and N66 [35].

### 3.3. Glycosylation on Noroviruses

HBGA is essential for norovirus infection. Fucosyltransferase 2 (FUT2), an enzyme that catalyzes 1,2-fucosylation of terminal galactose, regulates the production of HBGAs in intestinal epithelial cells. Individuals who lack FUT2s do not express HBGAs on their epithelial cells, rendering them exceptionally resistant to the gastroenteritis caused by certain norovirus strains, such as GII genotype 4 (Figure 1). Several pandemic GII.4 variations have been identified, including GII.4 US95/96, GII.4 Farmington Hills2002, GII.4 Hunter2004, GII.4 Yerseke2006a, GII.4 Den Haag2006b, GII.4 New Orleans2009, and GII.4 Sydney2012 [21,73]. Recently, non-GII.4 genotypes, including the GII.17 variant and the GII.2[P16] recombinant strain, have also been reported to cause epidemics [74,75]. The GII.17 variant caused norovirus outbreaks in some Asian countries, replacing the GII.4 Sydney2012 [74]. Moreover, the GII.2[P16] recombinant strain is reported to have swept through Japan, China, and Germany [75,76,77]. Despite the fact that HBGA glycosylation significantly alters binding affinity, little is revealed about the *N*- and *O*-glycosylation of norovirus capsid protein VP1, which merits additional exploration. On the other hand, the deamidation of Asn373 and the formation of isoD373 on the norovirus capsid protein VP1 impair its recognition of HBGAs [11] (Figure 1). Asn373 is found in the antigenic loop next to the HBGA binding site. Asn373 interacts with the glycan ligand through two direct hydrogen bonds; when converted to isoD373, only one hydrogen bond remains [78]. In addition, the peptides that contain isoD373 in the P dimmer domain do not show elevated flexibility [79]. Thus, the formation of isoD373 decreases the binding affinity of the P protein for HBGAs.

## 4. *O*-GlcNAcylation

### 4.1. O-GlcNAcylation on Proteins

**Figure 2:**
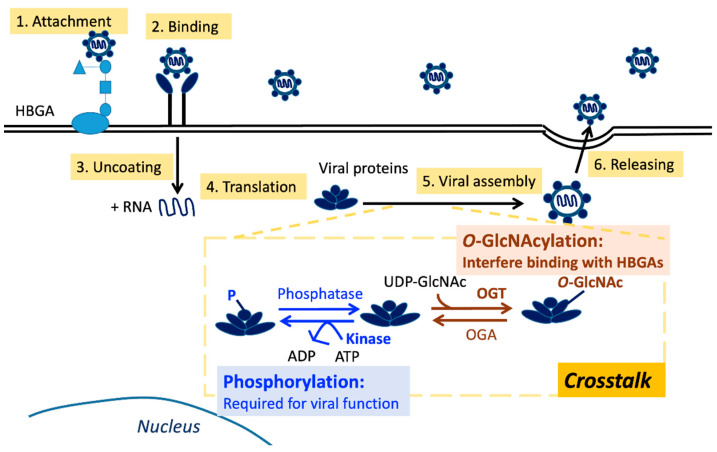
Replication cycle of noroviruses and post-translational modification crosstalk between phosphorylation and *O*-GlcNAcylation. Co-occurring PTMs on proteins are common, and PTM crosstalk between phosphorylation and *O*-GlcNAcylation is the most common since they will compete for the same residues (serine/threonine residues). Phosphorylation is required for viral function. In contrast, *O*-GlcNAcylation of norovirus capsid protein VP1 could interfere with receptor binding. Studies on the *O*-GlcNAcylation of noroviruses are few and lack direct evidence from receptor binding assays. Abbreviations: HBGA, histo-blood group antigen; VP1, major capsid protein VP1; ATP, adenosine triphosphate; ADP, adenosine diphosphate; OGT, *O*-linked *N*-acetylglucosamine (GlcNAc) transferase; OGA, *O*-GlcNAcase; *O*-GlcNAc, *O*-linked *N*-acetylglucosamine; UDP-GlcNAc, uridine diphosphate *N*-acetylglucosamine.

*O*-GlcNAcylation is a type of noncanonical glycosylation whereby *O*-linked *N*-acetylglucosamine (*O*-GlcNAc) is coupled to the hydroxyl groups of serine or threonine residues in proteins [82,83]. The hexosamine biosynthetic pathway, which incorporates glucose, amino acids, fatty acids, and nucleotide metabolism, produces the donor sugar for *O*-GlcNAcylation, UDP-GlcNAc. *O*-GlcNAc transferase (OGT) and *O*-GlcNAcase (OGA) catalyze the addition and removal of *O*-GlcNAc, respectively [84] (Figure 2). These two enzymes are found in all multicellular organisms and are substantially conserved from worms to humans [85]. In contrast to glycosylation, which is stable and localizes mainly at the ER and Golgi apparatus, *O*-GlcNAcylation is reversible and occurs in the cytoplasm [82]. *O*-GlcNAcylation has been implicated in several biological activities, including transcription, translation, metabolism, signal transmission, and apoptosis [84].

*O*-GlcNAcylation can be detected using various techniques such as lectins, antibodies, or click chemistry-based approaches. Lectins, such as Concanavalin A wheat germ agglutinin (WGA), are primarily used for binding to sialic acids and terminal β-GlcNAc on complex glycans [86,87,88]. Metabolic or chemical labeling followed by conjugation to an affinity linker, such as biotin or streptavidin, is a valuable method for detecting *O*-GlcNAcylation when combined with MS. In addition, some specific enzymes, such as galactosyltransferase, can selectively label the modified sites with a ketone-containing galactose analog, which also helps to identify this modification [87].

### 4.2. O-GlcNAcylation on Viruses

Unlike *N*- and *O*-glycosylation, which appears on the surface of viruses, *O*-GlcNAcylation occurs on proteins surrounding the nucleic acid components of viruses [85]. For example, multiple sites on the basic phosphoprotein of human cytomegalovirus are *O*-GlcNAcylated (Table 1) [50]. Furthermore, *O*-GlcNAcylation occurs in rotaviruses, where *O*-GlcNAc has been detected in RNA polymerase II transcription factors [47]. This modification is also present in adenoviruses and insect viruses, such as baculoviruses [48]. The implications of *O*-GlcNAcylation include playing a regulatory function, stabilizing multiprotein complexes, and conferring proteolytic resistance [82]. There have been few investigations of viral protein *O*-GlcNAcylation; nevertheless, the roles of *O*-GlcNAcylation in viruses are worth investigating further.

### 4.3. O-GlcNAcylation on Noroviruses

Most enveloped viruses have glycosylated surface proteins; however, only a few nonenveloped viruses have glycoproteins in their capsids. Noroviruses fall within the latter category. In 2022, several potential modification sites were discovered to be adjacent to the amino acid of the S domain (Thr65, Ser67) and P domain (Thr238, Ser519 in the P1 domain, and Thr350, Thr369, Thr371, Thr381 in the P2 domain), which may be relevant for receptor interactions [46] (Figure 2). The modifications were obtained by MALDI-MS of ethylaminylated peptides from the noroviral VP1 or by LC–MS2 sequencing on the native glycopeptides. Using immunoassays with lectins and antibodies, the authors confirmed the *O*-GlcNAcylation on VP1 protein. Based on this research, we speculate that *O*-GlcNAcylation may affect the binding affinity of noroviruses on the HBGAs. Several studies have revealed an interaction between *O*-GlcNAcylation and phosphorylation [85,89]. However, we lack sufficient studies on *O*-GlcNAcylation, although there are sufficient reports on norovirus phosphorylation.

## 5. Phosphorylation

### 5.1. Phosphorylation vs. O-GlcNAcylation

**Figure 3:**
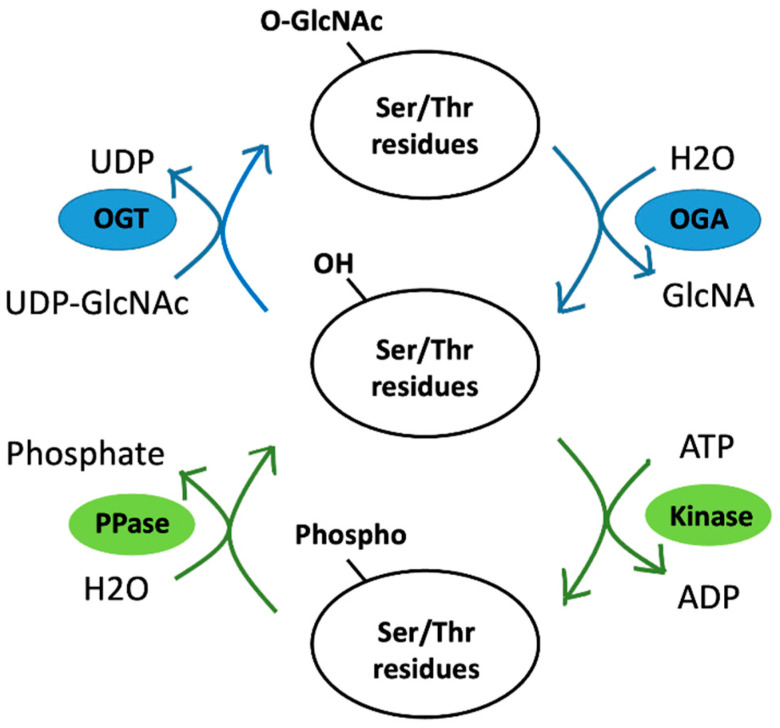
PTM crosstalk between *O*-GlcNAcylation and phosphorylation. Phosphorylation occurs when protein kinase attaches a phosphate group from ATP to the substrate; in contrast, PPase removes a phosphate group from a phosphoprotein. Similarly, OGT adds *O*-GlcNAc from UDP-GlcNAc to the substrates for *O*-GlcNAcylation. Conversely, OGA removes *O*-GlcNAc. These two processes are both reversible. Because phosphorylation and *O*-GlcNAcylation can compete for serine and threonine residues, PTM crosstalk occurs [83]. Abbreviations: *O*-GlcNAc, *O*-linked β-*N*-acetylglucosamine; OGT, *O*-GlcNAc transferase; OGA, *O*-GlcNAcase; Ser, serine; Thr, threonine; ATP, adenosine triphosphate; ADT, adenosine diphosphate; UPD, uridine diphosphate; PPase, protein phosphatase.

Protein phosphorylation is a well-known primary reversible switch for cell signaling control that plays an essential role in various cellular processes. Unlike *O*-GlcNAcylation, which OGT and OGA control, phosphorylation uses a myriad of protein kinases to transfer γ-phosphate from adenosine triphosphate (ATP) to the amino acid residue in the substrate protein (Figure 3). The phosphorylation of substrate proteins may occur at one or more sites. Nine amino acids are used as phosphate acceptors, including serine, threonine, and tyrosine (which contain hydroxyl groups (-OH), basic histidine, arginine, lysine, and acidic aspartic acid, glutamic acid, and cysteine. *O*-phosphorylation of serine, threonine, or tyrosine residues forms a phosphodiester (P-O) link between the -OH and the γ-phosphate of ATP. *N*-phosphorylation of the histidine, arginine, or lysine residues forms a phosphoramidite (P-N) link between the -NH and the γ-phosphate of ATP. *O*-phosphorylation is stable. In contrast, *N*-phosphorylation is acid-labile and, consequently, difficult to detect [90]. Adding a phosphate group to an amino acid residue substantially alters the protein structure. Phosphorylation affects protein characteristics such as enzymatic activity, stability, subcellular localization, and interaction with binding partners [22,91].

One of the most common approaches for detecting phosphorylation is to use radioactive-isotope-labeled substrates such as P32 orthophosphate. Furthermore, Western blot analysis and arrays have been used to detect phosphorylation. However, these techniques cannot provide information about the phosphorylation sites [59]. Mass spectrometry, coupled with enrichment techniques, has proven to be more robust in identifying PTM substrates and mapping PTM locations [59,63]. Because the overall proteome contains only a small fraction of phosphorylated proteins/peptides, enrichment is an essential step in MS detection of phosphorylation. Antibody-based affinity enrichment and ionic-interaction-based enrichment are the two enrichment procedures [59,92]. The use of pan-PTM antibodies to identify PTM peptides has been proven effective for tyrosine phosphorylation [88]. Furthermore, the interaction between the phosphate group and immobilized metal ions or titanium dioxide (TiO_2_) is the most common enrichment technique for analyzing phosphorylated peptides by MS, which recognizes over 3000 unique phosphopeptides [59,93,94,95].

As the density of co-occurring PTMs on proteins is high, several PTMs can affect the action of another via a process termed PTM crosstalk [83]. The most documented form is the PTM crosstalk between phosphorylation and *O*-GlcNAcylation (Figure 3). As they occur primarily on the same amino acid residues (serine and threonine), these two PTMs undergo crosstalk. Crosstalk may happen in various ways, including competition for the same site/residue (reciprocal crosstalk) and modifications affecting each other (proximal or distal to the peptide sequences) [83]. Furthermore, interruption of phosphorylation events alters the GlcNAcylation levels and vice versa. These findings demonstrate crosstalk between the two modifications [83,89].

### 5.2. Phosphorylation on Viruses

Many intracellular obligatory pathogens require phosphorylation to initiate a productive infection cycle [22]. The phosphorylation of viral proteins affects viral–host interactions, which substantially impact viral infection, replication, and cytotoxicity [30]. The PTMs of viral proteins, particularly RdRps, are common. Hepatitis C virus (HCV) RdRp, for example, is phosphorylated by protein kinase C-related kinase 2 (PRK2), which is essential for effective viral replication [91,96]. The viral RdRp enzyme is the main enzyme involved in viral RNA genome replication in plus-strand RNA viruses, including noroviruses [97]. RdRp phosphorylation has been proposed to be functionally connected to viral replication [16,98].

### 5.3. Phosphorylation on Noroviruses

Phosphorylation is widely recognized for directly regulating viral protein activity and acting as a molecular signal for a binding partner. Norovirus RdRp and FCV VPg proteins are reportedly phosphorylated. RdRp is phosphorylated at a position (Thr33) at the interface of the RdRp finger and thumb domains. This modification is exclusive to the most common norovirus genotypes, including GII.4 and GII.b [16]. The phosphorylation sites of the FCV VPg protein are threonine at position 80 and serine at position 107. These polymerases and virus-encoded proteins are required for viral function. RdRp, found in viral particles, is responsible for viral genome transcription and replication. VPg interacts with NS7 to help viral RNA synthesis, whereas its interaction with eIF4A triggers viral protein synthesis and VP1 functioning in viral encapsidation [15,99]. Both RdRp and VPg play essential roles in viral evolution and fitness. Consequently, the phosphorylation of RdRp and VPg may provide a mechanism for noroviruses and FCV, respectively, to regulate the viral life cycle and impact viral pathogenicity.

## 6. Conclusions

PTMs enable viruses to regulate molecular functions by maintaining stability, interacting with receptors, and suppressing the immune system. Many RNA viruses feature PTMs, as shown by previous studies; however, the understanding of norovirus PTMs remains poorly elucidated. Research suggests that phosphorylation promotes norovirus pathogenicity; however, information on glycosylation and *O*-GlcNAcylation is limited. Investigating the factors behind this conclusion is worthwhile because phosphorylation and *O*-GlcNAcylation frequently interact. One reason for this might be that only a few nonenveloped viruses exhibit glycosylation and *O*-GlcNAcylation. Furthermore, the potential for *O*-GlcNAcylation varies from cell to cell. The host cell type should be considered when analyzing viral glycosylation, and sophisticated mass spectrometry tools can aid the investigation. Given that glycosylation, *O*-GlcNAcylation, and phosphorylation are all associated with viral pathogenicity, identifying the modification sites on noroviruses will aid in developing future vaccines and treatments.

## Figures and Tables

**Table 1:** Established roles of *N*- and *O*-glycosylation, *O*-GlcNAcylation, and phosphorylation in viral pathogenesis.

PTM	Effect	Virus	Method	Year	Reference
*N*- and *O*-glycosylation	Attachment and entry	*N*-glycosylation: Ebola, HIV-1; *N*- and *O*-glycosylation: SARS-CoV2	PNGase F, Endo H_F_, HILIC-UPLC, MALDI TOF	2009, 2010, 2010, 2022	[32,33,34,35]
	Viral replication and maturation	*N*-glycosylation: influenza, DENV, rotavirus; *N*- and *O*-glycosylation: SARS-CoV2	PNGase F, Western blot, HILIC-UPLC, MALDI TOF	2022, 1999 2017, 2023	[35,36,37,38]
	Viral pathology	*N*-glycosylation: DENV, influenza, ZIKV	PNGase F, Western blot, lectins	1999, 2017 2023	[36,37,39]
	Immune evasion by glycan shielding	*N*-glycosylation: Ebola, influenza, coronavirus, HIV-1, arenavirus, SARS-CoV2	PNGase F, Western blot, Synapt G2S, Orbitrap MS, HILIC-UPLC	2009, 2017 2020, 2016 2015, 2022 2022	[32,37,40,41,42,43,44]
	Release of new virus particles	*N*-glycosylation: influenza; *N*- and *O*-glycosylation: SARS-CoV2	PNGase F, LC-MS	2022 1999	[35,45]
*O*-GlcNAcylation	Attachment and entry into cells	Norovirus	Lectins, antibodies, GC-MS	2022	[46]
	RNA polymerase II transcription factors	Rotavirus	Enzyme	1991	[47]
	Viral protein stability	Adenovirus, baculovirus	WGA, [14C] GlcN radiolabeled fiber	1992 1989	[48,49]
	Viral pathology	Human cytomegalovirus	Electrospray-MS	1994	[50]
Phosphorylation	Viral replication and maturation	Alphavirus, Ebola, HCV	Antibodies, Western blot	2022, 2018 2019	[51,52,53]
	Viral protein synthesis	Norovirus, FCV	Electrophoresis	2016, 2011	[15,16]
	Inhibition of immune pathways	DENV, ZIKV, yellow fever virus, SARS-CoV	Metabolic labeling immunoblot	2005 2009	[54,55]
	Release of new virus particles	Lassa virus	Gel electrophoresis LC-MS/MS	2018	[56]

Effects of the three types of PTMs. Abbreviations: PTMs, post-translational modifications; HIV-1, human immunodeficiency virus-1; DENV, dengue virus; ZIKV, Zika virus; HCV, hepatitis C virus; FCV, feline calicivirus; SARS-CoV, severe acute respiratory syndrome-associated coronavirus; PNGase F, peptide-*N*-glycosidase F; Endo H_F_, endo-β-*N*-acetylglucosaminidase H; HILIC, hydrophilic interaction liquid chromatography; UPLC, ultraperformance liquid chromatography; MS, mass spectrometry; GC, gas chromatography; MALDI TOF, matrix-assisted laser desorption/ionization-time of flight; SYNAPT G2S high definition mass spectrometer (HDMS) is used for discovery and targeted omic-based experiments; Orbitrap MS, ion trap high-resolution mass spectrometry analyzer; WGA, wheat germ agglutinin; GalNAc, *N*-acetyl-P-D-galactosamine; MS, mass spectrometry.

## Data Availability

The data that support the findings of this study are available in the material of this article.

## Abbreviations

DENV	Dengue virus
DPAGT1	Dolichol phosphate-dependent *N*-acetylglucosamine 1-phospho-transferase
ER	Endoplasmic reticulum
Endo H	Endoglycosidase H
Endo H_F_	Endo-β-*N*-acetylglucosaminidase H
FCV	Feline calicivirus
FUT2	Fucosyltransferase 2
GalNAc	*N*-acetylgalactosamine
GC-MS	Gas chromatography-mass spectrometry
HBGA	Histo-blood group antigen
HCV	Hepatitis C virus
HILIC	Hydrophilic interaction liquid chromatography
HIV-1	Human immunodeficiency virus-1
HuNoV	Human norovirus
LC-MS	Liquid chromatography-mass spectrometry
MBL	Mannose-binding lectin
MNV	Murine norovirus
MS	Mass spectrometry
MSE	Data-independent collection mode mass spectrometry
NS	Nonstructural protein
*O*-GlcNAc	*O*-linked *N*-acetylglucosamine
OGA	*O*-GlcNAcase
OGT	*O*-GlcNAc transferase
ORF	Open reading frame
OST	Oligosaccharyltransferase
PLGS	Protein Lynx Global Server
PNGase F	Peptide-*N*-glycosidase F
PTM	Post-translational modification
RdRp	RNA-dependent RNA polymerase
RNA	Ribonucleic acid
SARS-CoV	Severe acute respiratory syndrome-associated coronavirus
UDP-GlcNAc	Uridine diphosphate *N*-acetylglucosamine
UPLC	Ultraperformance liquid chromatography
WGA	Wheat germ agglutinin
ZIKV	Zika virus
